# A composite indicator of derived neutrophil–lymphocyte ratio and lactate dehydrogenase correlates with outcomes in pancreatic carcinoma patients treated with PD-1 inhibitors

**DOI:** 10.3389/fonc.2022.951985

**Published:** 2022-10-25

**Authors:** Shiyun Chen, Shiyuan Guo, Miaomiao Gou, Yuting Pan, Mengjiao Fan, Nan Zhang, Zhaoli Tan, Guanghai Dai

**Affiliations:** ^1^ Medical School of Chinese People’s Liberation Army, Beijing, China; ^2^ Department of Oncology, The First Medical Centre, Chinese People’s Liberation Army General Hospital, Beijing, China

**Keywords:** pancreatic cancer, immune checkpoint inhibitors, overall survival, progression-free survival, derived neutrophil–lymphocyte ratio, lactate dehydrogenase

## Abstract

**Background:**

There are currently no established biomarkers that can predict whether advanced pancreatic carcinoma (PC) patients would benefit from immune checkpoint inhibitors (ICIs). Our study investigated whether the pretreatment composite biomarker of derived neutrophil–lymphocyte ratio (dNLR) and lactate dehydrogenase (LDH) can be used as a reliable prognostic factor for the survival of PC patients receiving PD-1 inhibitor therapy.

**Methods:**

Patients with advanced PC treated with PD-1 inhibitors at a single center from September 2015 to September 2020 were included. The high levels of dNLR (≥3) and LDH (≥250 U/L) were considered to be risk factors. Based on these two risk factors, patients in this study were categorized into two risk groups: the good dNLR-LDH group, without risk factors, and the intermediate/poor dNLR-LDH group, with one to two risk factors. Overall survival (OS) and progression-free survival (PFS) served as this study’s primary and secondary endpoints. Cox regression models were used to identify independent prognostic factors for survival benefit.

**Results:**

There were 98 patients in our study. The good group included 61 (62.2%) patients and the intermediate/poor group included 37 (37.8%). The overall patients with PC who received immunotherapy had a median OS of 12.1 months, and the good dNLR-LDH group had a significantly longer OS compared with the intermediate/poor dNLR-LDH group (44.2 vs. 6.4 months; p < 0.010); median PFS was 3.7 and 2.5 months (p = 0.010). The number of metastatic sites >2 and immunotherapy as third-line or later was associated with worse PFS, and the line of immunotherapy and the dNLR-LDH indicator were independent prognostic factors for OS, according to multivariate analysis.

**Conclusion:**

The pretreatment composite biomarker of dNLR and LDH can be used as a prognostic biomarker in patients with advanced PC treated with PD-1 inhibitors.

## Introduction

Pancreatic carcinoma (PC) is one of the deadliest malignant tumors with a low survival rate. Until now, the prognosis remains dismal; the overall 5-year survival rate is less than 5% (1). The main factor for the low survival rate is the late presentation of most patients. Since the disease is relatively asymptomatic at the initial stage, most patients are not examined and treated until the late stage. Not more than 20% of newly diagnosed PC patients have the opportunity to undergo surgery. The tumor is highly malignant and progresses rapidly; chemotherapies and palliative care are still the most essential treatment for PC patients. However, the efficacy of existing drugs is limited (2, 3). The improved chemotherapy regimen FOLFIRINOX significantly increased adverse reactions but only slightly prolonged the median survival time (4).

Although no adequate treatment has been found yet, immunotherapy has gradually become a promising new therapy for PC (5, 6) and other malignancies (7, 8). However, there is still a notable portion of patients with PC that has poor curative effects after receiving this treatment. Therefore, it is critical to identify people who are candidates for immunotherapy and can benefit from it.

Convenient prognostic biomarkers can play a significant role in clinical practice. Previously, Mezquita et al. developed the lung immune prognostic index (LIPI) by combining baseline derived neutrophil–lymphocyte ratio (dNLR) and lactate dehydrogenase (LDH) to classify patients with non-small cell lung cancer treated with programmed death-1 (PD-1)/programmed death ligand-1 (PD-L1) inhibitors (9), thus providing a new way to predict prognosis.

The predictive usefulness of dNLR in combination with LDH in a range of solid tumors treated with immunotherapy, including renal cell carcinoma, non-small cell lung cancer, and gastric cancer, has been confirmed in several early studies (10–14). Therefore, we analyzed real-world data to assess the prognostic value of the pretreatment composite biomarker of dNLR and LDH in PC patients treated with PD-1 inhibitors.

### Patients and methods

Patients with advanced PC who received PD-1 inhibitors (nivolumab/pembrolizumab/sintilimab) at the Chinese PLA General Hospital (Beijing, China) between September 2015 and September 2020 were included in this study. The inclusion criteria were as follows (1): age > 18 years old (2); Eastern Cooperative Oncology Group (ECOG) performance status of 0–1; (3) diagnosed as locally advanced or advanced PC; and (4) available CT scans and blood test results during immunotherapy.

Demographic, clinical, and pathological data were collected, including age, gender, type of PD-1 inhibitor, smoking history, smoking years, differentiation degree of tumor tissue, number of metastatic sites, prior target therapy, whether PD-1 inhibitor was combined with other drugs, line of immunotherapy, baseline CA19-9 level, white blood cell count, absolute neutrophil count, absolute lymphocyte count, and serum LDH level. This research was authorized by the Ethics Committee of the Chinese PLA General Hospital and performed according to the principles of the Declaration of Helsinki.

The dNLR-LDH indicator was calculated by dNLR (absolute neutrophil count/[white blood cell count − absolute neutrophil count]) and LDH. The cutoff values of dNLR and LDH were 3 and the upper limit of normal value (ULN, 250 U/L), respectively. Patients were divided into the good dNLR-LDH group (dNLR < 3 and LDH normal) and the intermediate/poor dNLR-LDH group (intermediate: dNLR < 3 and LDH ≥ ULN, or dNLR ≥ 3 and LDH < ULN; poor: dNLR ≥ 3 and LDH ≥ ULN).

According to the Response Evaluation Criteria in Solid Tumors (RECIST 1.1), the efficacy of immunotherapy was evaluated. Overall survival (OS) was defined as the time from the first PD-1 inhibitor treatment to death. Progression-free survival (PFS) was defined as the time from the first PD-1 inhibitor treatment to progressive disease (PD) or death.

### Statistical analysis

IBM SPSS version 26.0 was used to perform statistical analysis. The Kaplan–Meier method was utilized to analyze OS and PFS, and the differences were evaluated by log-rank test. The clinical characteristics of patients were compared by Chi-square or Fisher’s exact test. The Cox proportional hazard regression model was used for univariate and multivariate analysis. Multivariate analysis was performed on covariates that showed a significant correlation with OS and PFS in univariate analysis. All statistical tests were two-sided, and *p* < 0.05 was statistically significant.

## Results

### Clinical characteristics of patients

**Figure 1:**
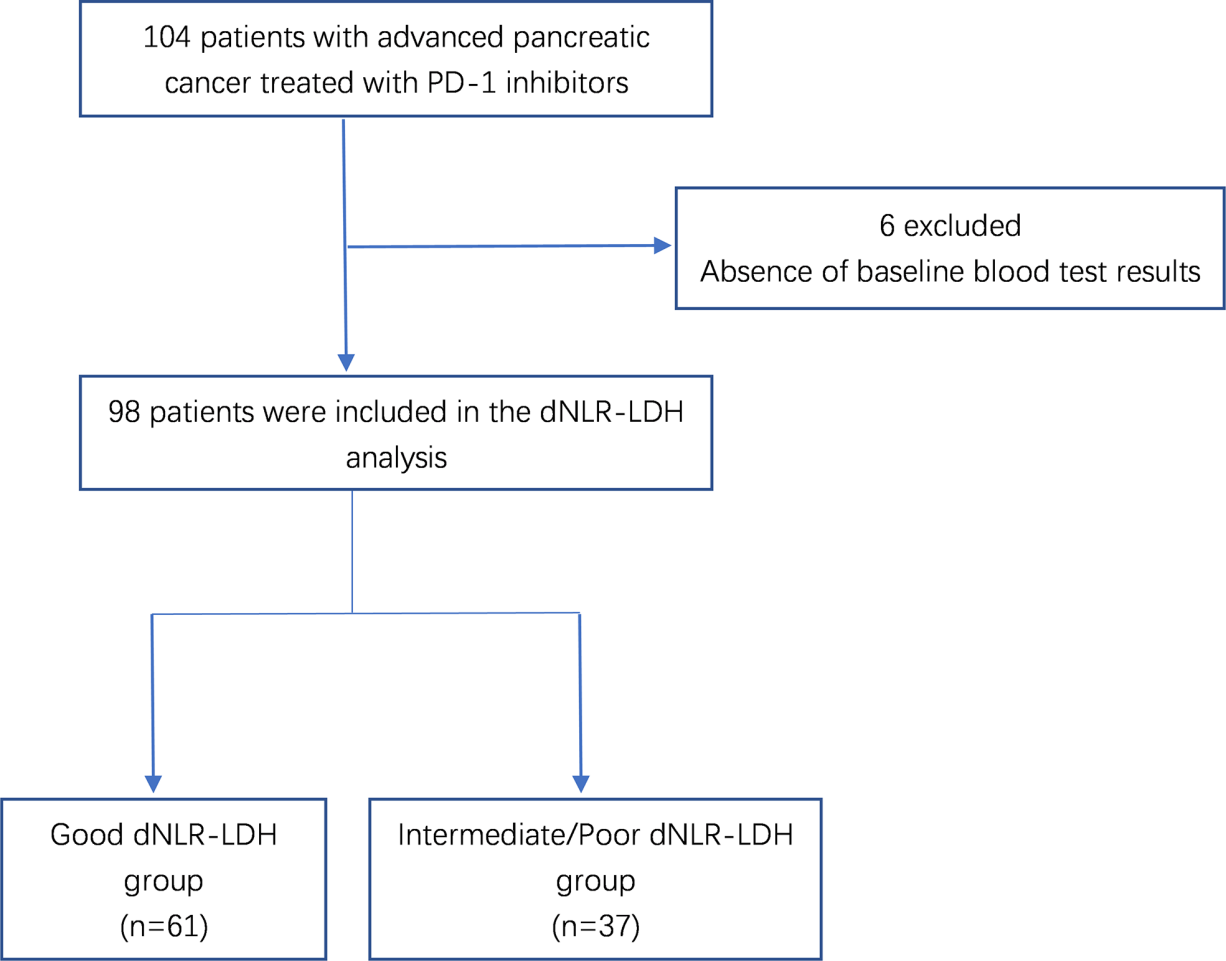
Flowchart of patient selection.

A total of 104 patients with advanced PC were treated with PD-1 inhibitors. After excluding 6 patients without relevant data required for dNLR-LDH grouping, 98 PC patients were included in the study for clinical data analysis (**Figure 1**). Most patients (92.9%) received PD-1 inhibitors combined with chemotherapy or targeted therapy. The median age was 56 years, and 71.4% were male; 57.1% of patients had low differentiation and 85.7% of patients had ≤2 metastatic sites. Patients with baseline CA19-9 level higher than normal accounted for 69.4%. A total of 61 patients (62.2%) had a good dNLR-LDH indicator. Detailed baseline clinicopathological features of patients are summarized in **Tables 1** and **2**.

**Table 1:** Characteristics of patients with advanced PC.

Characteristics	No. of Patients (*n* = 98)	Percentage (%)
Age, years		
≥60	32	32.7
<60	66	67.3
Sex		
Male	70	71.4
Female	28	28.6
PD-1 inhibition agent		
Nivolumab	33	33.7
Pembrolizumab	9	9.2
Sintilimab	56	57.1
Smoking history		
Smoke	36	36.7
Never smoke	62	63.3
Smoking exposure, pack-years		
≤30	83	84.7
>30	15	15.3
Histological differentiating degree		
Low differentiation	56	57.1
Moderate and high differentiation	42	42.9
Metastatic sites		
0–2	84	85.7
>2	14	14.3
Baseline CA19-9		
≤ULN	23	23.5
>ULN	68	69.4
Missing	7	7.1
Prior target therapy		
Yes	8	8.2
No	86	87.8
Missing	4	4.1
Combined with other drugs		
Yes	91	92.9
No	7	7.1
Line of immunotherapy		
1st	75	76.5
2nd	16	16.3
3rd or above	7	7.1
LDH		
≥ULN	7	7.1
<ULN	91	92.9
dNLR		
≥3	34	34.7
<3	64	65.3
dNLR-LDH indicator		
Good	61	62.2
Intermediate/poor	37	37.8

PD-1, programmed cell death-1; LDH, lactate dehydrogenase; ULN, upper limit of normal; dNLR, derived neutrophil-to-lymphocyte ratio.

**Table 2:** Differences of patients’ characteristics between the good dNLR-LDH group and the intermediate/poor dNLR-LDH group.

Characteristics	No. of Patients (%)		*p*-value
	Good dNLR-LDH	Intermediate/Poor dNLR-LDH	
	(*n* = 61)	(*n* = 37)	
Age, years			
≥60	22 (36.1)	10 (27.0)	0.355
<60	39 (63.9)	27 (73.0)	
Sex			0.469
Male	42 (68.9)	28 (75.7)	
Female	19 (31.1)	9 (24.3)	
PD-1 inhibition agent			0.321
Nivolumab	19 (31.1)	14 (37.8)	
Pembrolizumab	4 (6.6)	5 (13.5)	
Sintilimab	38 (62.3)	18 (48.6)	
Smoking history			0.543
Smoke	21 (34.4)	15 (40.5)	
Never smoke	40 (65.6)	22 (59.5)	
Smoking exposure, pack-years			0.398
≤30	50 (82.0)	33 (89.2)	
>30	11 (18.0)	4 (10.8)	
Histological differentiating degree			0.229
Low differentiation	32 (52.5)	24 (64.9)	
Moderate and high differentiation	29 (47.9)	13 (35.1)	
Metastatic sites			0.106
0–2	55 (90.2)	29 (78.4)	
>2	6 (9.8)	8 (21.6)	
Baseline CA19-9			0.839
≤Normal level	14 (24.6)	9 (26.5)	
>Normal level	43 (75.4)	25 (73.5)	
Missing	4	3	
Prior target therapy			0.52
Yes	4 (7.0)	4 (10.8)	
No	53 (93.0)	33 (89.2)	
Missing	4	0	
Combined with other drugs			0.773
Yes	57 (93.4)	34 (91.9)	
No	4 (6.6)	3 (8.1)	
Line of immunotherapy			0.959
1st	47 (77.0)	28 (75.7)	
2nd	10 (16.4)	6 (16.2)	
3rd or above	4 (6.6)	3 (8.1)	

PD-1, programmed cell death-1; ULN, upper limit of normal; LDH, lactate dehydrogenase; dNLR, derived neutrophil-to-lymphocyte ratio.

### Association between dNLR-LDH and prognosis

#### Effect of the dNLR-LDH indicator on OS

**Figure 2:**
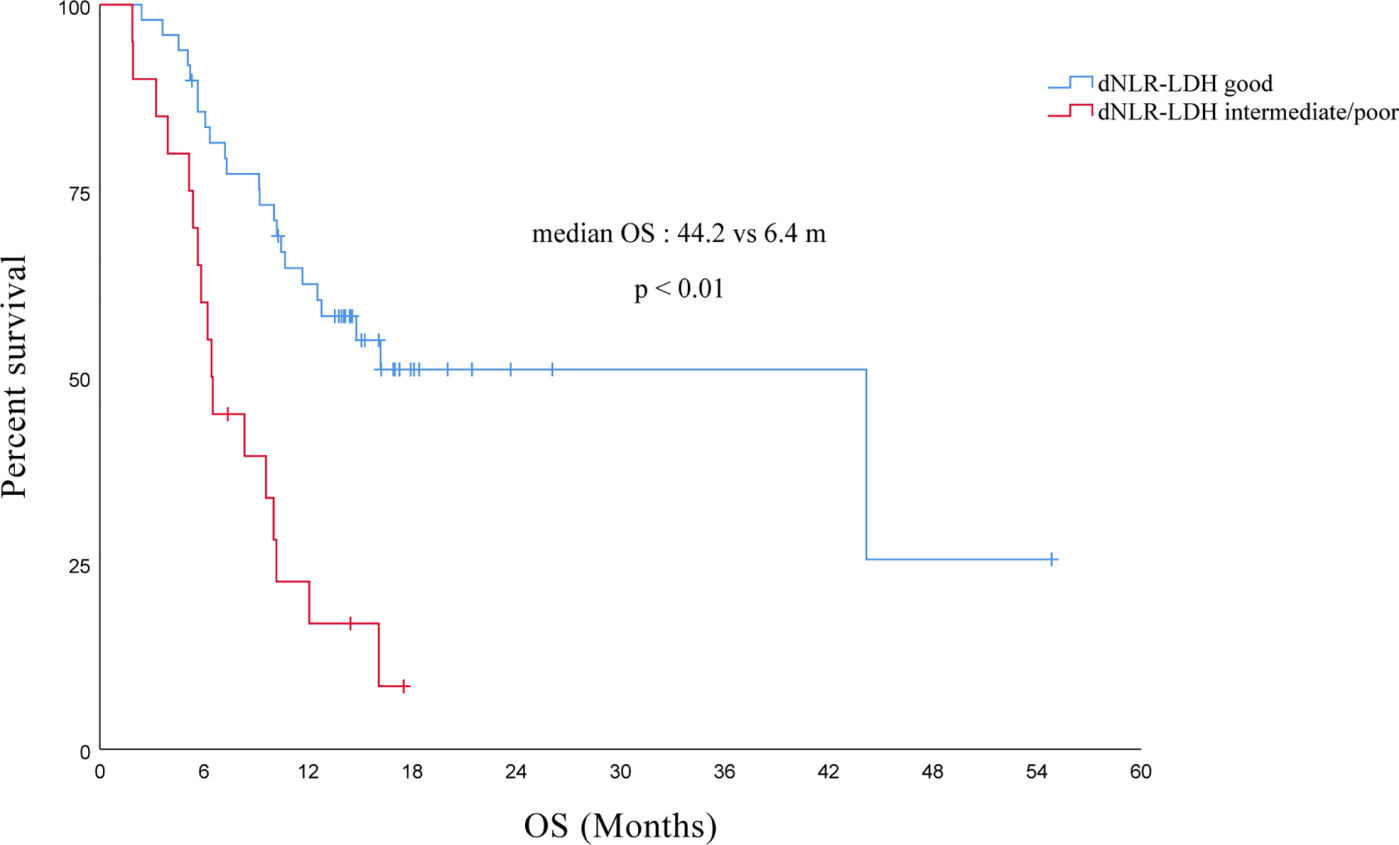
Association between pretreatment dNLR-LDH indicator with OS.

The median OS of all PC patients treated with immunotherapy (single/combined) was 12.1 (95% CI, 7.4–16.8) months. Compared with the intermediate/poor dNLR-LDH group, the OS of the good dNLR-LDH group was significantly prolonged (*p* < 0.001) (**Figure 2**). The median OS in the good and intermediate/poor dNLR-LDH groups was 44.2 (95% CI, 9.4–79.0) and 6.4 (95% CI, 5.8–7.1) months, respectively.

In univariate analysis, the number of metastatic sites, previous targeted therapy, gender, line of immunotherapy, types of PD-1 inhibitors, and the dNLR-LDH indicator were significantly correlated with OS (*p* < 0.05). Multivariate analysis showed that intermediate/poor dNLR-LDH was associated with a significantly increased risk of death (HR, 2.52; 95% CI, 1.23–5.13; *p* = 0.011). The line of immunotherapy ≥3 is also an independent poor prognostic factor (**Table 3**).

**Table 3:** Univariate and multivariate analysis for OS in PC patients treated with ICIs.

		Univariate		Multivariate		
Variable	Category	HR (95% CI)	p-value	HR (95% CI)	p-value
Gender	Male vs. Female	2.645 (1.166–5.999)	0.020	1.592 (0.662–3.828)	0.299
Age	≥60 vs. <60 years	1.097 (0.587–2.052)	0.771		
Smoking	Yes vs. No	1.361 (0.720–2.571)	0.343		
Smoking exposure	>30 vs. ≤30 years	1.224 (0.542–2.766)	0.627		
Histological differentiating degree	Moderate and High vs. Low	0.652 (0.344–1.233)	0.188		
Metastatic sites	>2 vs. 0–2	2.511 (1.151–5.479)	0.021	1.049 (0.386–2.850)	0.926
Baseline CA19-9	>ULN vs. ≤ULN	1.438 (0.627–3.297)	0.391		
Prior target therapy	Yes vs. No	8.502 (3.198–22.604)	0.000	1.168 (0.236–5.791)	0.849
Combined with other drugs	Yes vs. No	0.810 (0.272–2.415)	0.706		
Line of immunotherapy	≥3rd vs. 2nd vs. 1st	3.670 (2.369–5.687)	0.000	2.671 (1.403–5.082)	0.003
PD-1 inhibition agent	Nivolumab vs. Pembrolizumab vs. Others	0.525 (0.352–0.783)	0.002	0.615 (0.340–1.113)	0.108
dNLR-LDH indicator	Intermediate/poor vs. Good	3.415 (1.784–6.535)	0.000	2.515 (1.232–5.132)	0.011

OS, overall survival; ICI, immune checkpoint inhibitor; PD-1, programmed cell death-1; ULN, upper limit of normal; LDH, lactate dehydrogenase; dNLR, derived neutrophil-to-lymphocyte ratio.

#### Effect of the dNLR-LDH indicator on PFS

**Figure 3:**
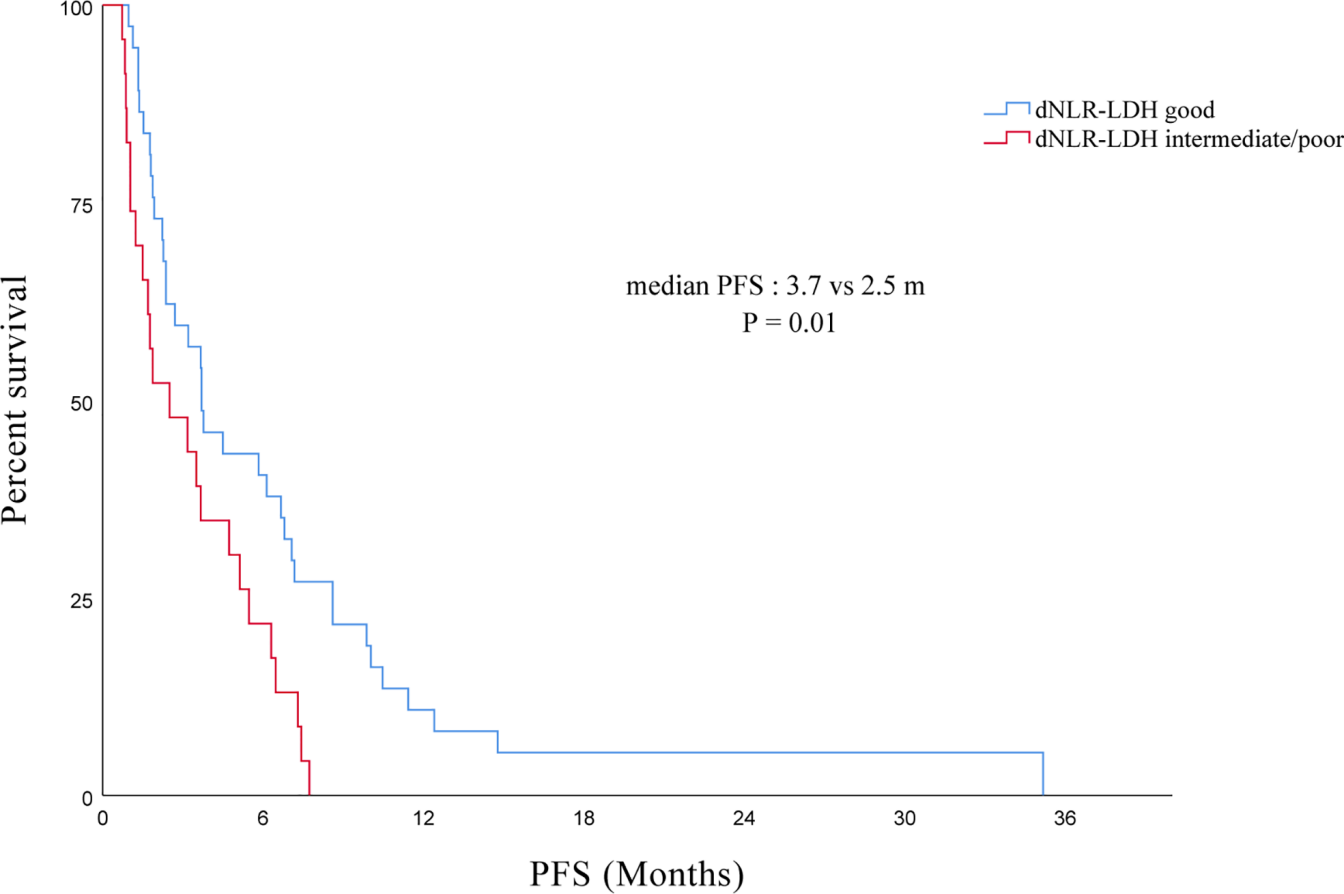
Association between pretreatment dNLR-LDH indicator with PFS.

The median PFS of all PC patients treated with immunotherapy (single/combined) was 3.5 (95% CI, 2.4–4.6) months. The PFS of the good dNLR-LDH group was longer than that of the intermediate/poor dNLR-LDH group (*p* = 0.010) (**Figure 3**). The median PFS in the good and intermediate/poor dNLR-LDH groups was 3.7 (95% CI, 2.2–5.3) and 2.5 (95% CI, 0.3–4.7) months, respectively.

In univariate analysis, the degree of histological differentiation, the number of metastatic sites, the line of immunotherapy, and the dNLR-LDH indicator were significantly correlated with PFS (*p* < 0.05). Multivariate analysis showed that the number of metastatic sites and the line of immunotherapy ≥3 were the adverse factors affecting PFS (*p* < 0.05). The dNLR-LDH indicator was another independent determinant, with a marginal *p* = 0.067 (HR, 1.76; 95% CI, 0.96–3.22) (**Table 4**).

**Table 4:** Univariate and multivariate analysis for PFS in PC patients treated with ICIs.

		Univariate			Multivariate	
Variable	Category	HR (95% CI)	p-value		HR (95% CI)	p-value
Gender	Male vs. Female	1.713 (0.964–3.043)	0.067			
Age	≥60 vs. <60 years	0.798 (0.469–1.358)	0.405			
Smoking	Yes vs. No	1.314 (0.780–2.214)	0.305			
Smoking exposure	>30 vs. ≤30 years	0.968 (0.474–1.977)	0.929			
Histological differentiating degree	Moderate and High vs. Low	0.463 (0.267–0.803)	0.006		0.636 (0.359–1.125)	0.120
Metastatic sites	>2 vs. 0–2	3.913 (1.826–8.383)	0.000		2.302 (1.010–5.248)	0.047
Baseline CA19-9	>ULN vs. ≤ULN	1.795 (0.935–3.446)	0.079			
Prior target therapy	Yes vs. No	1.531 (0.607–3.863)	0.367			
Combined with other drugs	Yes vs. No	0.801 (0.335–1.913)	0.617			
Line of immunotherapy	≥3rd vs. 2nd vs. 1st	2.353 (1.518–3.649)	0.000		1.999 (1.265–3.158)	0.003
PD-1 inhibition agent	Nivolumab vs. Pembrolizumab vs. Others	0.705 (0.470–1.057)	0.091			
dNLR-LDH indicator	Intermediate/poor vs. Good	2.058 (1.173–3.611)	0.012		1.759 (0.961–3.220)	0.067

PFS, progression-free survival; ICI, immune checkpoint inhibitor; PD-1, programmed cell death-1; ULN, upper limit of normal; LDH, lactate dehydrogenase; dNLR, derived neutrophil-to-lymphocyte ratio.

## Discussion

Immunotherapy is a popular topic in the current medical community and has promising prospects in a variety of cancers. However, not all patients, particularly those with PC, can benefit from PD-1 inhibitor therapy. We need to find out patients who respond to immunotherapy. Therefore, it is crucial to explore biomarkers that can predict treatment prognosis.

Several studies have previously demonstrated the predictive value of the composite biomarker of dNLR and LDH for immunotherapy prognosis in various solid tumors (10–12), which can provide practical guidance for clinical work. The dNLR-LDH indicator, which can be inferred from patient blood test results, has the advantages of being affordable, easily reproducible, and widely available. Monitoring these indicators can early predict the prognosis of some advanced pancreatic cancer patients receiving PD-1 inhibitor therapy, and are able to select and stratify patients who may benefit, facilitating the development of more precise and individualized treatment.

To our knowledge, this is the first study to show that pretreatment dNLR-LDH can serve as a prognostic predictive biomarker in PC patients receiving PD-1 inhibitor therapy. dNLR can reflect the inflammatory state of the whole body. It was calculated as absolute neutrophil count/[white blood cell count − absolute neutrophil count]. It has been implicated in cancer progression through cytokine production, and it can also promote immune evasion (15, 16). It is well known that systemic inflammation is closely associated with the prognosis of immunotherapy. Indicators of inflammation potentially presented in the blood can indicate poor prognosis in patients with malignancies, including elevated levels of neutrophils, platelets, NLR, platelet–lymphocyte ratio (PLR), and LDH (17, 18). dNLR can provide more information than NLR because it includes monocytes and other granulocyte subsets in addition to lymphocytes; it is considered an unfavorable prognostic factor in a wide range of malignancies (19, 20), whereas LDH can reflect the patient’s tumor burden. Several studies have shown the correlation between dNLR and LDH and the prognosis of immunotherapy (18, 21–23), and in previous studies of non-small cell lung cancer, patients with a “good” LIPI score (i.e., dNLR < 3 and normal LDH) had better OS and PFS, regardless of whether they have received immunotherapy, targeted therapy, or chemotherapy (12). To our knowledge, there are no studies that have explored the relationship between the combined indicator of dNLR and LDH and the prognosis of pancreatic cancer treatment. However, there have been some studies showing that NLR can be used as a prognostic indicator in pancreatic cancer patients receiving chemotherapy (24). Furthermore, high LDH levels at baseline may be associated with shorter OS in patients with advanced pancreatic cancer treated with chemotherapy (25). Therefore, we can predict that dNLR-LDH may not only be a prognostic marker for pancreatic cancer patients receiving PD-1 inhibitors, but could also play an important role in the early prediction for the prognosis of other treatment options (e.g., chemotherapy). This speculation remains to be further confirmed by more studies.

In univariate analysis, our findings imply that the pretreatment dNLR-LDH indicator was associated with PFS and OS in ICI-treated advanced PC patients. The multivariate analysis showed that the pretreatment dNLR-LDH indicator is an independent prognostic factor for OS (*p* < 0.05). However, in the correlation analysis of PFS, marginal *p* = 0.067. We must take into account the limits of this retrospective study: We cannot arbitrarily define the results as not statistically significant. Since PFS is affected by several factors, such as the time and frequency of tumor evaluation, radiological assessments were performed within a time range of ±2 weeks. From mPFS, we can also see that patients with a good dNLR-LDH indicator had a better prognosis, with an mPFS of 3.7 (95% CI, 2.2–5.3) and 2.5 (95% CI, 0.3–4.7) months in the good and intermediate/poor dNLR-LDH groups, respectively. In addition, although many factors were considered in the multivariate analysis, we failed to incorporate some factors into the study that may also affect the final results because some tests in patients with PC were not routinely performed in clinical work, resulting in missing test reports, such as PD-L1 and tumor mutation burden (TMB) (26). However, recent studies have shown that the predictive value of the composite biomarker of dNLR and LDH is independent of PD-L1 expression and TMB (27, 28). Furthermore, in multivariate analysis, our study also found that the line of immunotherapy and the number of metastatic sites were associated with the PFS of PC patients treated with immunotherapy, and the line of immunotherapy is also related to the patient’s OS. The number of metastasis sites reflected the tumor burden of patients; in other words, the heavier the tumor load, the higher the possibility of short-term progress after receiving immunotherapy. If patients who had already undergone a lengthy course of treatment before received third-line immunotherapy or later, their overall condition at this point may be worse than those who received first-line immunotherapy. This may be one of the reasons why the use of immunotherapy as a third-line or later treatment is associated with worse PFS and OS.

There are some shortcomings within our investigation. First, this is a single-center retrospective study with a limited sample size, which could not avoid possible confounding factors and selective bias. Due to the small sample size, we divided the cohort into two groups (good dNLR-LDH and intermediate/poor dNLR-LDH) instead of three (good, intermediate, and poor dNLR-LDH) for analysis. Nonetheless, our study provides a simple and non-invasive method to help identify patients with advanced PC who might benefit from PD-1 inhibitor therapy in clinical practice.

## Conclusion

Our finding confirms the utility of the dNLR-LDH indicator in the prognostic prediction of advanced PC patients treated with PD-1 inhibitors, and their potential to help with patient stratification and clinical decision. Further prospective studies of the dNLR-LDH indicator in anti-PD-1-based randomized clinical trials are warranted in the future.

## Data availability statement

The raw data supporting the conclusions of this article will be made available by the authors, without undue reservation.

## Ethics statement

This study was reviewed and approved by The Ethics Committee of Chinese PLA General Hospital. The patients/participants provided their written informed consent to participate in this study.

## Author contributions

SC was in charge of writing and analysis. GD and ZT provided the guide and idea. SG, MG, YP, MF, and NZ contributed to analysis. All authors contributed to the article and approved the submitted version.

## Conflict of interest

The authors declare that the research was conducted in the absence of any commercial or financial relationships that could be construed as a potential conflict of interest.

## Publisher’s note

All claims expressed in this article are solely those of the authors and do not necessarily represent those of their affiliated organizations, or those of the publisher, the editors and the reviewers. Any product that may be evaluated in this article, or claim that may be made by its manufacturer, is not guaranteed or endorsed by the publisher.

## Abbreviations

dNLR, derived neutrophil–lymphocyte ratio; ECOG, Eastern Cooperative Oncology Group; ICIs, immune checkpoint inhibitors; LDH, lactate dehydrogenase; LIPI, lung immune prognostic index; OS, overall survival; PC, pancreatic carcinoma; PD-L1, programmed death ligand-1; PD-1, programmed death-1; PFS, progression-free survival; PD, progressive disease; TMB, tumor mutation burden; ULN, upper limit of normal.
